# Exosomes derived from mature chondrocytes facilitate subcutaneous stable ectopic chondrogenesis of cartilage progenitor cells

**DOI:** 10.1186/s13287-018-1047-2

**Published:** 2018-11-21

**Authors:** Yahong Chen, Ke Xue, Xiaodie Zhang, Zhiwei Zheng, Kai Liu

**Affiliations:** 1 Department of Plastic and Reconstructive Surgery, Shanghai Ninth People’s Hospital, College of Stomatology, Shanghai Jiao Tong University School of Medicine, 639 ZhiZaoJu Road, Shanghai, 200011 People’s Republic of China; 2 Department of Oral and Maxillofacial-Head and Neck Oncology, Shanghai Ninth People’s Hospital, College of Stomatology, Shanghai Jiao Tong University School of Medicine, 639 ZhiZaoJu Road, Shanghai, 200011 People’s Republic of China; 3 National Clinical Research Center for Oral Diseases, 639 ZhiZaoJu Road, Shanghai, 200011 People’s Republic of China; 4 Shanghai Key Laboratory of Stomatology & Shanghai Research Institute of Stomatology, 639 ZhiZaoJu Road, Shanghai, 200011 People’s Republic of China

**Keywords:** Cartilage, Progenitor/stem cells, Exosomes, Chondrocytes, Bone mesenchymal stem cells, Chondrogenesis

## Abstract

**Background:**

Developing cartilage constructed with the appropriate matrix composition and persistent chondrogenesis remains an enduring challenge in cartilage defects. Cartilage progenitor cell (CPC)-based tissue engineering has attracted recent attention because of its strong chondrogenic differentiation capacity. However, due to the lack of a suitable chondrogenic niche, the clinical application of CPC-regenerated cartilage in the subcutaneous environment remains a challenge. In this study, exosomes derived from chondrocytes (CC-Exos) were used to provide the CPC constructs with a cartilage signal in subcutaneous environments for efficient ectopic cartilage regeneration.

**Methods:**

Rabbit CPC-alginate constructs were prepared and implanted subcutaneously in nude mice. CC-Exos were injected into the constructs at the same dose (30 μg exosomes per 100 μL injection) after surgery and thereafter weekly for a period of 12 weeks. Exosomes derived from bone mesenchymal stem cells (BMSC-Exos) were used as the positive control. The mice in the negative control were administered with the same volume of PBS. At 4 and 12 weeks after implantation, the potential of CC-Exos and BMSC-Exos to promote chondrogenesis and stability of cartilage tissue in a subcutaneous environment were analyzed by histology, immunostaining, and protein analysis. The influences of BMSC-Exos and CC-Exos on chondrogenesis and angiogenic characteristics in vitro were assessed via coculturing with CPCs and human umbilical vein endothelial cells.

**Results:**

The CC-Exos injection increased collagen deposition and minimized vascular ingrowth in engineered constructs, which efficiently and reproducibly developed into cartilage. The generated cartilage was phenotypically stable with minimal hypertrophy and vessel ingrowth up to 12 weeks, while the cartilage formed with BMSC-Exos was characterized by hypertrophic differentiation accompanied by vascular ingrowth. In vitro experiments indicated that CC-Exos stimulated CPCs proliferation and increased expression of chondrogenesis markers while inhibiting angiogenesis.

**Conclusions:**

These findings suggest that the novel CC-Exos provides the preferable niche in directing stable ectopic chondrogenesis of CPCs. The use of CC-Exos may represent an off-the-shelf and cell-free therapeutic approach for promoting cartilage regeneration in the subcutaneous environment.

## Background

The structural and functional repair of sizeable subcutaneous cartilage defects remains a challenge in plastic and reconstructive surgery [1, 2]. Strategies for cartilage defects include autologous chondrocytes implantation and matrix-assisted chondrocyte implantation [3, 4]. While successful in some respects, each of these therapies has limitations, including donor limitation, donor morbidity, and degradation of the graft tissue [5, 6]. In recent years, stem cell-based cartilage tissue engineering has shown great promise [7]. Cartilage progenitor/stem cells (CPCs), which are considered an attractive cell source, have been increasingly investigated in cartilage regeneration because of their strong chondrogenic potential [8–10]. Unfortunately, the lack of a suitable chondrogenic niche still hinders efficient and stable ectopic chondrogenesis of progenitor cells in subcutaneous environments [11, 12].

Indeed, the tissue regeneration niche plays a crucial role in determining the ultimate phenotype of implanted stem cells [13–15]. Chondrocytes are one of the major niche cell types in cartilage and play an essential role in the maintenance of the cartilage microenvironment. Many studies have demonstrated the improved chondrogenesis of stem cells after coculturing with chondrocytes [1, 16]. Chondrocytes can also create a proper chondrogenic niche for stabilizing the chondrogenic phenotype of stem cells in ectopic cartilage regeneration [17]. Furthermore, it is hypothesized that the chondrocytes empower the chondrogenic efficacy of progenitor cells mainly through the paracrine effects of trophic factors [16, 18, 19].

Among the numerous factors secreted, exosomes have been identified as the principal agent in mediating the therapeutic efficacy of endogenous or grafted cells in several disease indications [20, 21]. Exosomes are nanosized (30–200 nm), bi-lipid membrane vesicles secreted by most cell types. Exosomes have been found to contain various types of bioactive microRNAs, nucleic acids, proteins, and unique gene products [22–24]. Additionally, it was reported that exosomes could transfer trophic factors to neighboring cells and serve as mediators of intercellular communication [25]. In contrast to the conventional cell-based therapy, the use of exosomes is advantageous from the perspectives of off-the-shelf and cell-free regenerative medicine approach and the ease of minimally invasive injection.

However, it is still unknown whether chondrogenesis can be enhanced by exosomes derived from chondrocytes (CC-Exos). Moreover, the interaction between CC-Exos and cartilage hypertrophy has not been elucidated. Here, the central hypothesis that CC-Exos promotes cartilage regeneration and stabilizes the cartilage tissue phenotype via the inhibition of angiogenesis is tested using a CPC-based subcutaneous cartilage regeneration model. In addition, the exosomes derived from bone mesenchymal stem cells (BMSC-Exos) have now been used for in situ cartilage defect repair with robust cartilage tissue formation [21, 26]. BMSC-Exos were recently reported to accelerate neo-tissue filling and enhance matrix synthesis of type II collagen and sulfated glycosaminoglycan [27–30]. In this study, BMSC-Exos were used as the positive control to test the null hypothesis that there is no difference between CC-Exos and BMSC-Exos in cartilage regeneration.

## Methods

### Isolation and culture of CPCs, BMSCs, and chondrocytes

CPCs in the auricular cartilage tissue of rabbits (provided by Shanghai Chuansha Breeding Factory, *n* = 6) were harvested via differential adhesion to fibronectin as described previously [31, 32]. Briefly, cells were seeded onto 100-mm plastic petri dishes (pretreated with 10 μg/mL fibronectin overnight at 37 °C) in low-glucose Dulbecco’s modified Eagle’s medium (DMEM, Gibco, USA). After 20 min, the cells were rinsed twice with phosphate-buffered saline (PBS) and cultured in low-glucose DMEM containing 10% fetal bovine serum (FBS, Gibco, USA). BMSCs and chondrocytes from rabbits were isolated and expanded according to previously established methods [31, 33]. The isolated cells were cultured in DMEM supplemented with 10% FBS and expanded to passage 2 (P2) to extract exosomes.

### Preparation of CC-Exos and BMSC-Exos

After reaching 80% confluency, chondrocytes and BMSCs were rinsed with PBS and cultured with serum-free medium for 48 h. CC-Exos and BMSC-Exos were both isolated and purified from the conditioned medium following a previous protocol [34]. In detail, the obtained conditioned medium was centrifuged at 3000×*g* for 30 min at 4 °C, followed by filtering with a 0.45-μm and a 0.22-μm filter (SteritopTM, Millipore, USA) to remove the remaining cells and cellular debris. Finally, exosomes were isolated by size fractionation and concentrated 50× by centrifugation using an Ultra-clear tube (Millipore) with a molecular weight cutoff of 100 kDa. Exosomes were stored at − 80 °C for the following experiments. Nano-Sight (NS300, Malvern, England), transmission electron microscopy (TEM, JEOL microscope, JSM-7001TA, Tokyo, Japan), and Western blot were used to identify exosomes.

### Exosome labeling and exosome uptake studies

Isolated CC-Exos or BMSC-Exos were labeled with CM-Dil red fluorescent membrane linker dye (Invitrogen, Waltham, MA, USA) as previously described [35, 36]. Briefly, 1 μM cell-labeling solution was added to 200 μg exosomes suspended in 1 mL PBS and was incubated for 5 min at 37 °C and 15 min at 4 °C. Subsequently, the mixture was washed to remove unbound CM-Dil. CPCs were incubated with CM-Dil-labeled exosomes (30 μg/mL) for 12 h according to a previous study [21]. Then, cells were washed twice with PBS, fixed in 4% paraformaldehyde, and stained with phalloidin and DAPI. Finally, cells were observed under a Zeiss Confocal LSM 710 microscope (Carl Zeiss, Jena, Germany) to determine the uptake of the labeled exosomes.

### In vivo chondrogenesis of CPCs induced by exosome in subcutaneous non-chondrogenic sites

**Scheme 1:**
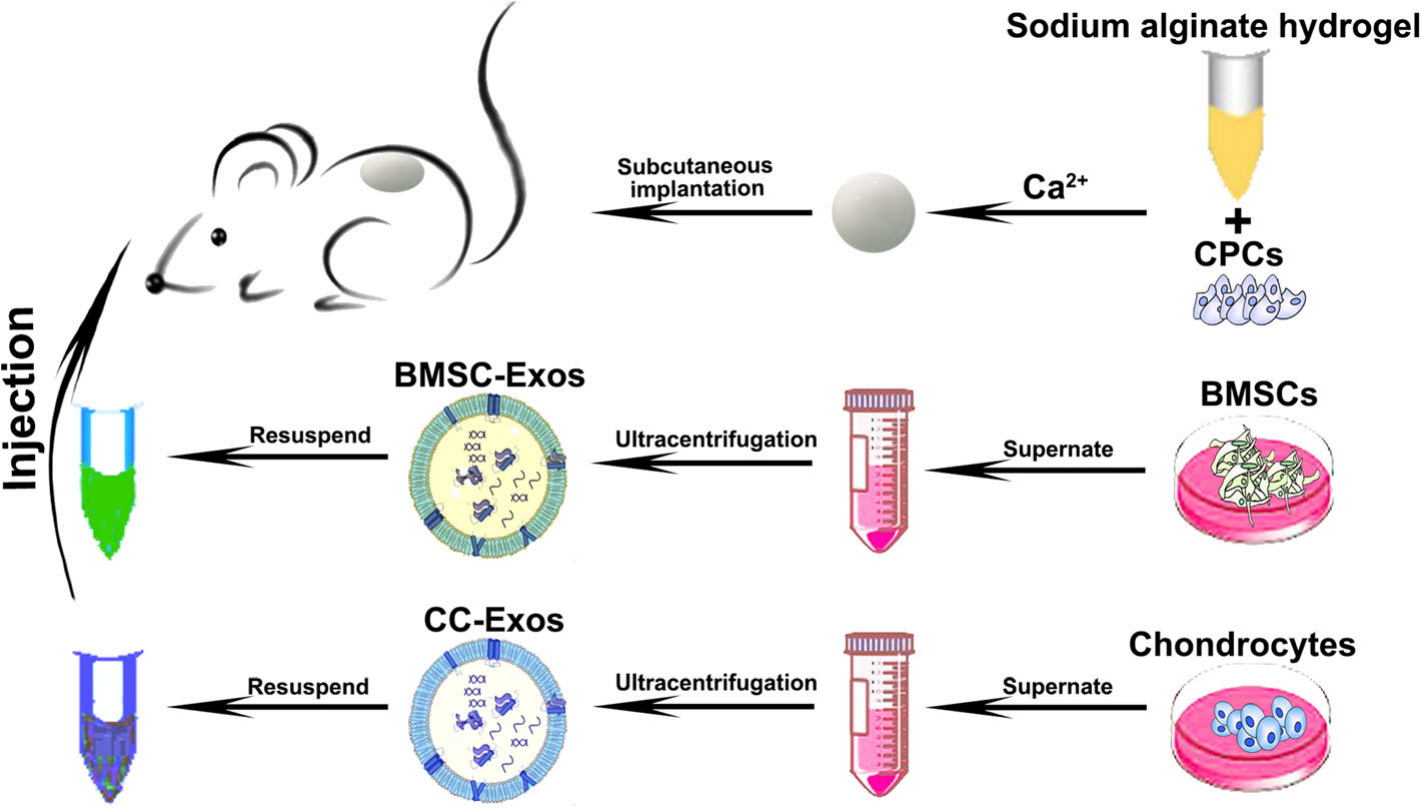
Schematic illustration of the preparation of exosomes and their applications in vivo

All procedures were approved by the Animal Research Committee of Shanghai Jiao Tong University Affiliated Ninth People’s Hospital. Implants were formed by encapsulating 1 million CPCs in 100 μL 1.5% (wt/vol) sodium alginate (Aladdin, China) using 100 mM CaCl_2_ [37]. The engineered tissues were implanted subcutaneously as previously reported [17, 37] in 30 female nude mice; each mouse was randomly assigned to receive a local injection of PBS, CC-Exos, or BMSC-Exos. Exos solutions in PBS were prepared under sterile conditions. CC-Exos and BMSC-Exos (30 μg exosomes per 100 μL injection) were administered subsequently on a weekly basis [38, 39]. Five injection sites evenly distributed in the construct were determined, and 20-μL solution was injected per site. The same volume of PBS was used as the negative control. The detailed process is shown in Scheme 1.

### Histology

At 4 weeks or 12 weeks, the samples were explanted and histologically analyzed. After gross observation, samples were fixed in 4% paraformaldehyde for 24 h. The samples were dehydrated with a graded alcohol series, embedded in paraffin, and sectioned perpendicularly to the implants into 5-μm-thick sections. Hematoxylin and eosin (H&E), safranin-O/fast green (S-F), and toluidine blue (T-B) were used for histological observations.

### Immunohistochemistry and immunofluorescence

Immunohistochemistry was performed as previously described [12, 27]. Dewaxed sections were washed in PBS, and endogenous peroxidase activity was quenched by immersion in 2% (*v*/*v*) hydrogen peroxide for 5 min. Antigen retrieval was carried out by incubating the sections with a sodium citrate buffer for 30 min. After additional washes in PBS, the sections were blocked with 1.5% goat serum for 30 min at room temperature followed by incubation with the primary antibody (COL II or COL X Abcam, Cambridge, UK) overnight at 4 °C. The sections were then incubated with a peroxidase-conjugated secondary antibody, visualized with a 3,3-diaminobenzidine solution (DAB Substrate Kit, Burlingame, CA, USA), and counter-stained with hematoxylin.

For immunofluorescence examination, the sections were incubated with primary antibodies (anti-CD31, Abcam) overnight at 4 °C. An Alexa Fluor 594-labeled secondary antibody was applied under light protection. The nuclei were counter-stained with 0.1 mg/mL DAPI, and the stained sections were examined using a Zeiss Confocal LSM 710 microscope (Carl Zeiss, Jena, Germany).

### Western blot

The protocol and procedure for Western blot were performed as described in previous reports [40]. For each sample, the 30 μg extracted total protein was loaded onto a 10–15% SDS/PAGE gel. The gel-separated protein was then transferred to a PVDF membrane (Millipore) and incubated with the primary antibodies of anti-transforming growth factor-β (TGF-β, Abcam), anti-SMAD2/3 (Abcam), anti-collagen type II (COL II, Abcam), anti-SOX-9 (Abcam), anti-collagen type X (COL X, Abcam), anti-Indian hedgehog (IHH, Affinity, OH, USA), anti-matrix metalloproteinase 13 (MMP 13, Affinity), anti-stem cell-derived factor 1 (SDF-1, Affinity), anti-vascular endothelial growth factor (VEGF, Affinity), and anti-β-actin (Invitrogen) at 37 °C for 2 h, followed by incubation with horseradish peroxidase-conjugated secondary antibodies. Protein expression was visualized, and the values were normalized against β-actin.

### Cell proliferation

The various effects of exosomes on the proliferation of CPCs were evaluated using a Cell Counting Kit-8 (CCK8, Dojindo Laboratories, Kumamoto, Japan). CPCs pretreated with 10 μg/mL or 30 μg/mL of the two different exosomes were seeded into 96-well plates, the media were changed every other day for 7 days, and cell proliferation curves were constructed by measuring with a microplate reader at a wavelength of 450 nm.

Ki67 staining was used to determine the effect of exosomes on CPC proliferation. CPCs were incubated with CM-Dil-labeled exosomes (10 μg/mL or 30 μg/mL) for 12 h, and the cells cultured without exosomes were used as the negative control. Then, cells were fixed with 4% formaldehyde for 30 min at room temperature, washed twice with PBS, and permeabilized with ice-cold methanol for 5 min at 4 °C. Blocking was performed with 5% goat serum for 30 min at 37 °C, followed by incubation with anti-Ki67 at a concentration of 1:1000 (Abcam) overnight at 4 °C, then washed twice with PBS. Incubation with anti-rabbit fluorescent-conjugated secondary antibody for 1 h at room temperature in the dark was then performed. CPCs were washed twice with PBS and stained with DAPI. Ki67, CM-Dil, and DAPI immunofluorescence images were captured using a confocal microscope.

### Quantitative real-time polymerase chain reaction

Total RNA was isolated and reverse transcription for cDNA synthesis was performed as previously described [21, 41]. Quantitative real-time PCR (qRT-PCR) was performed using a Power SYBR Green PCR Master Mix (Applied Biosystems) in a real-time thermal cycler (Stratagene, La Jolla, CA, USA). Glyceraldehyde 3-phosphate dehydrogenase (GADPH) was used as an internal control gene to normalize the expression of the other mRNAs. The results obtained after calibration with the GADPH expression level were calculated using the 2^−ΔΔCT^ method and presented as fold increases relative to the negative control. The primers for qRT-PCR analysis are listed in Table 1.

**Table 1:** Primers used for real-time RT-PCR

Genes	Sequence (5′-3′)
COL II	F: TCC TGT GCG ACG ACA TAA TCT; R: GCA GTG GCG AGG TCA GTA G
SOX-9	F: AGG TGC TCA AGG GCT ACG AC; R: TTG ACG TGG GGC TTG TTC T
VEGF	F: CCT TTG TGG TGG ACG CTA TC; R: CCG AAG TGA CTT GGG AAC TTG
SDF-1	F: ATC CTC AAC ACG CCC AAC TG; R: TGA CCC GCC TCT CAC ATC TT
GAPDH	F: ATG GTG AAG GTC GGA GTG A; R: AAC ATC CAC TTT GCC AGA GTT A

### In vitro migration assay

The migration effects of CPCs with various exosomes was evaluated using a transwell assay [42, 43]. CPCs were placed in the upper chamber of transwell inserts (Corning, NY, USA). Different culture medium was added in the lower chamber according to the group designation: negative control (NC)—DMEM medium with 1% FBS; positive control (PC)—DMEM medium with 10% FBS; CC-Exos group (CC-Exos)—DMEM medium with 1% FBS and 30 μg/mL CC-Exos; BMSC-Exos group (BMSC-Exos)—DMEM with 1% FBS and 30 μg/mL BMSC-Exos. After incubating at 37 °C for 6 h or 12 h, cells on the lower side of the insert filter were stained with phalloidin for F-actin and DAPI for nuclei, and the numbers of cells were counted. Migration of CPCs cultured with various exosomes was further evaluated using a scratch wound assay as described previously [23]. The cells that migrated from the original wound edge at 0 h, 12 h, and 24 h were counted from the photographs.

### Apoptosis

To assess the effect of different treatments on cell apoptosis, treated CPCs were doubly stained with Annexin V-FITC and PI and analyzed by flow cytometry. CPCs (1 × 10^5^ cells) from each group (CPCs cultured in different culture medium for 12 h; negative control (NC)—DMEM medium with 1% FBS; positive control (PC)—DMEM medium with 10% FBS; CC-Exos group (CC-Exos)—DMEM medium with 1% FBS and 30 μg/mL CC-Exos; BMSC-Exos group (BMSC-Exos)—DMEM with 1% FBS and 30 μg/mL BMSC-Exos) were incubated with FITC-labeled Annexin V and propidium iodide (BD Biosciences, San Jose, CA, USA) for 15 min at room temperature in the dark. The percentage of apoptotic cells was determined by flow cytometry (BD FACSCalibur, Beckman Coulter).

### In vitro angiogenesis assay

In vitro tubular formation assay was conducted using Matrigel (BD Bioscience, Oxford, UK). The Matrigel was coated onto 48-well plates at 37 °C for 30 min to complete gelation according to the manufacturer’s instructions. Human umbilical vein endothelial cells (HUVECs) were seeded into pretreated plates, and 1% FBS, 10% FBS, or 30 μg/mL of different exosomes were added to each well. After 3 h, cells were photographed using an inverted light microscope. Triplicate samples were tested for each condition, and five random microscopic images were collected for the measurement.

### Statistical analysis

Numerical data are presented as the mean ± standard deviation (SD) and were analyzed with a one-way ANOVA followed by Tukey’s post hoc test. Statistical analysis was performed using GraphPad Prism version 5.0 (GraphPad Software, San Diego, CA, USA). Among the various groups, *P* < 0.05 was considered to indicate a significant difference.

## Results

### Characterization of BMSC-Exos and CC-Exos

**Fig. 1:**
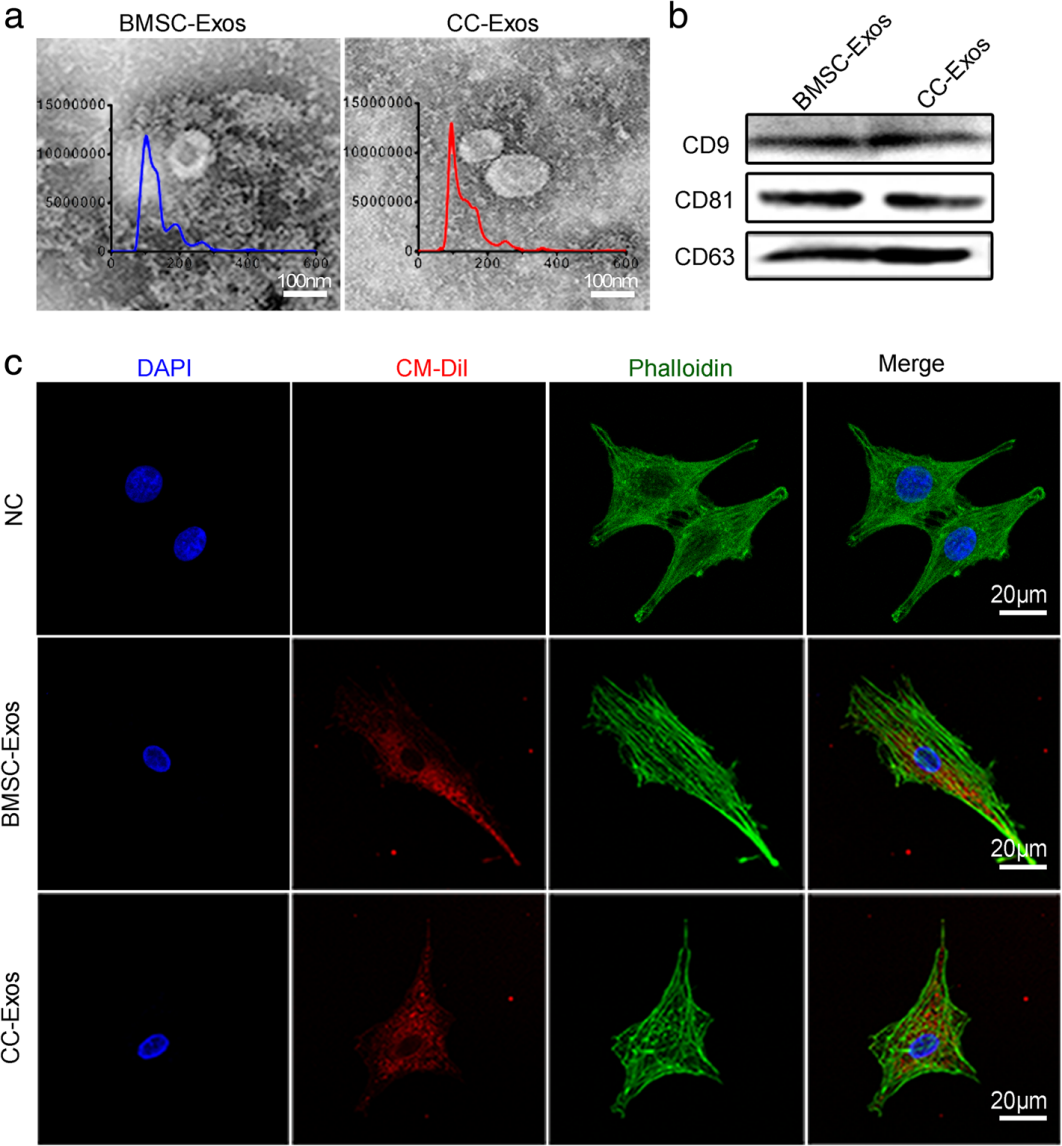
Characterization of BMSC-Exos and CC-Exos. **a** Nano-Sight measurement of exosome size and distribution and morphology of exosomes under transmission electron microscopy. Scale bars = 100 nm. **b** Western blot analysis of exosome-specific markers CD9, CD63, and CD81. **c** Representative immunofluorescence photomicrograph of CM-Dil (red)-labeled exosomes absorbed by CPCs at 12 h, the F-actin of which were stained by phalloidin (green), and the nuclei were stained by DAPI (blue). Scale bars = 20 μm

TEM clearly revealed that the exosomes purified from CCs and BMSCs both showed a cup-shaped or round-shaped form with a diameter of 30–200 nm, which was verified by the Nano-Sight analysis (Fig. 1a). Exosome-associated markers, CD9, CD63, and CD81, were shown by Western blot (Fig. 1b). The above results showed two kinds of exosomes were successfully isolated. In addition, fluorescence microscope images revealed CM Dil-labeled exosomes in the cytoplasm of the CPCs (Fig. 1c), confirming the successful internalization of both kinds of exosomes at 12 h.

### CC-Exos promotes ectopic cartilage regeneration

**Fig. 2:**
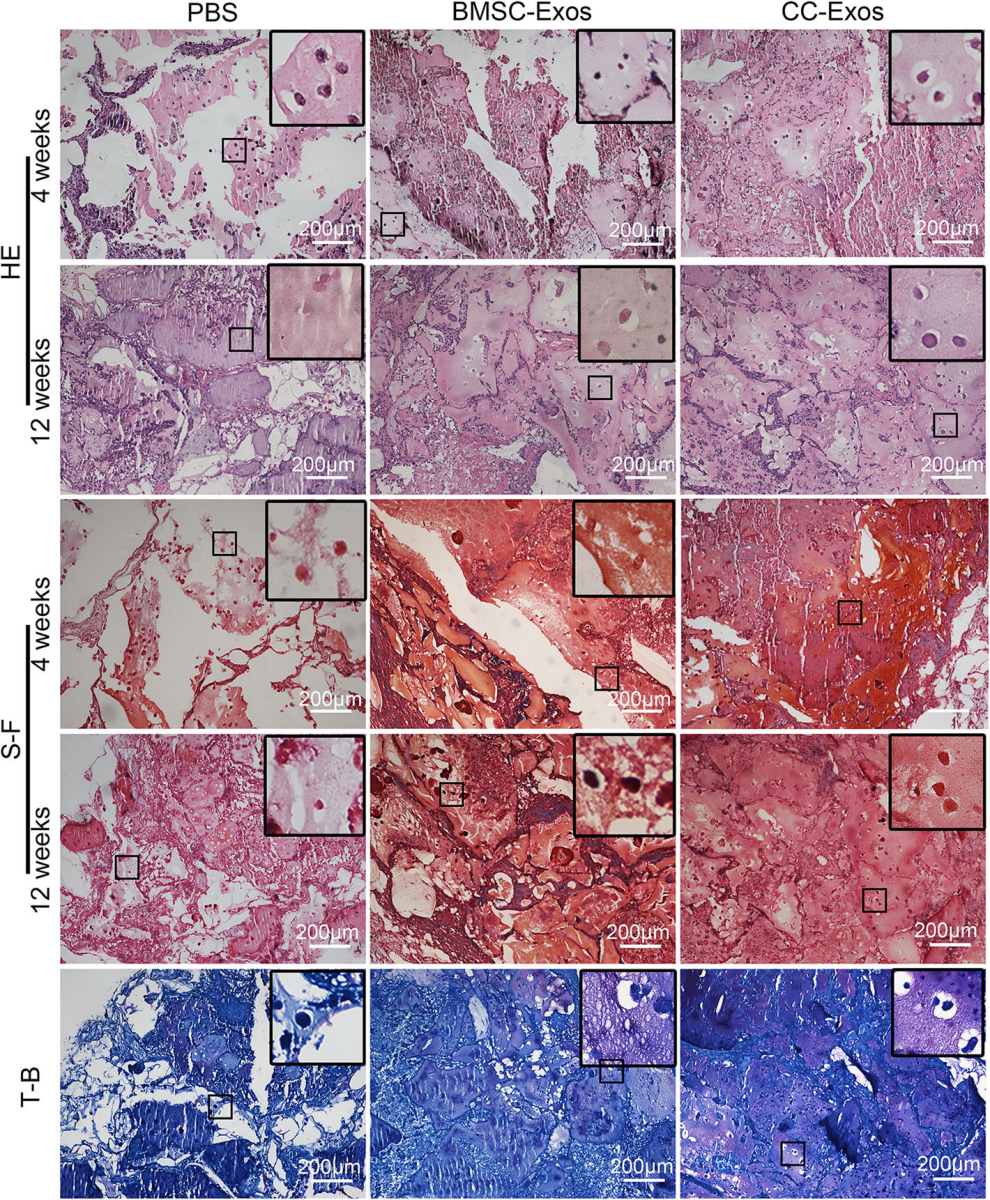
Histological analysis. H&E staining, safranin O/fast green (S-F) staining, and toluidine blue (T-B) staining in 4 weeks or 12 weeks showed enhanced ectopic cartilage formation in the BMSC-Exos and CC-Exos groups, compared to the PBS group. The enlarged images detailed the chondrocytes surrounded by a cartilaginous matrix, where more chondrocytes were found in the CC-Exos group, followed by BMSC-Exos and PBS groups. At week 4, the CPC constructs supplied with PBS did not lead to obvious cartilage matrix deposition. The addition of both BMSC-Exos and CC-Exos improved the matrix formation, compared with the PBS group. By 12 weeks, the BMSC-Exos and CC-Exos groups could maintain their cartilage-like appearance, and the histological results revealed significantly more contiguous cartilage matrix deposition than was observed at 4 weeks in each group. Black rectangles: typical chondrocytes, which are in high magnification. Scale bars = 200 μm

**Fig. 3:**
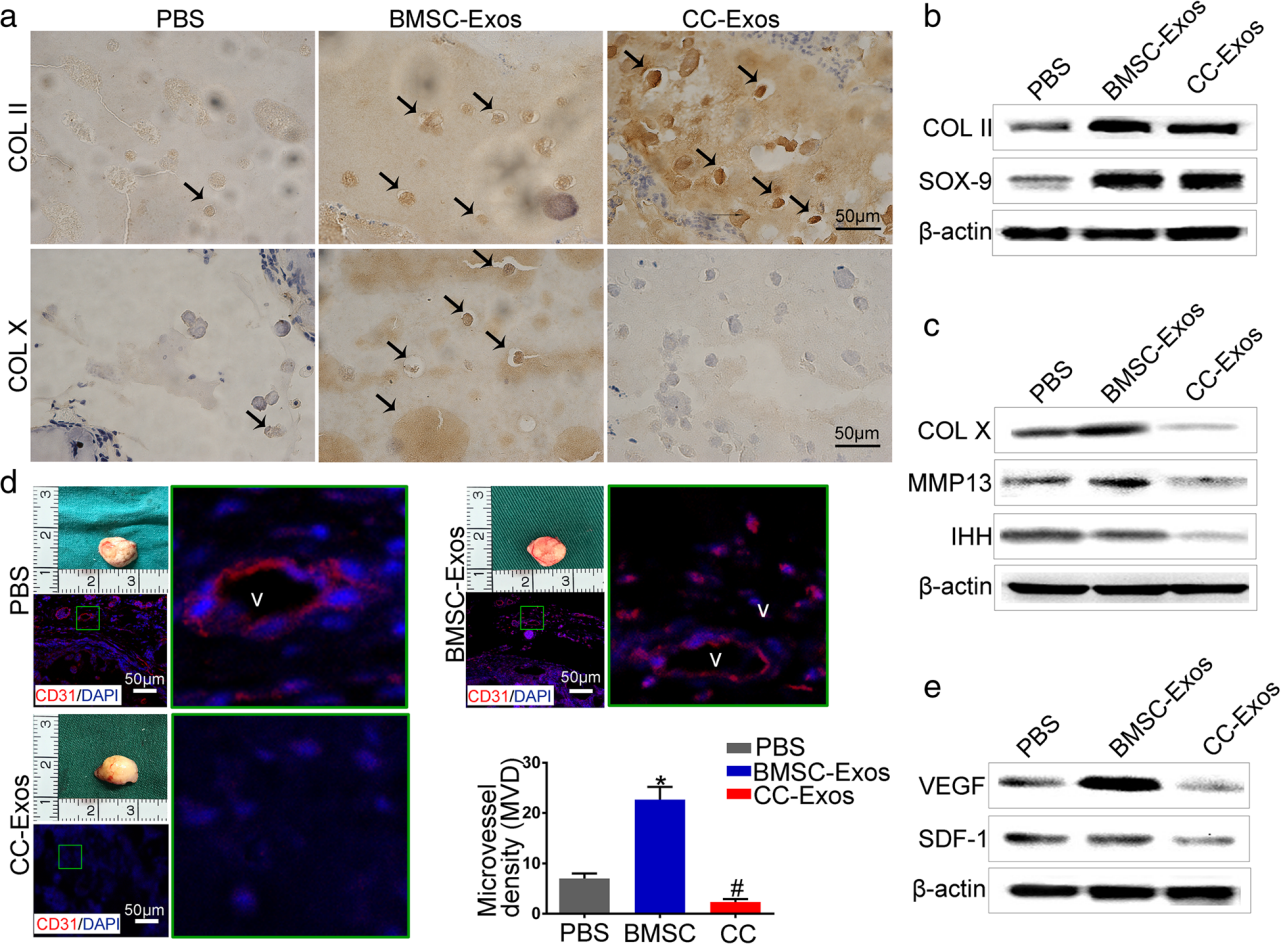
Cartilage regeneration and stability of the implants. **a** Representative image of immunohistochemical staining of cartilaginous matrix. The protein expression of COL II was increased in the BMSC-Exos and CC-Exos groups, compared to the PBS group. However, COL X is indicative of hypertrophy and was expressed in the BMSC-Exos group, indicating that several of the chondrocytes were exhibiting a hypertrophic phenotype. The black arrows point to the positively stained regions. Scale bars = 50 μm. **b**, **c** Western blot of Col II, SOX-9 (markers of chondrogenesis), and Col X, IHH, and MMP13 (markers of hypertrophy) secreted by the implants exposed to different exosomes and PBS. **d** Gross appearances and immunostaining of CD31 of in vivo implants at 12 weeks. The gross observation showed that the implants with CC-Exos did not lead to any obvious blood vessel formation. However, the BMSC-Exos induced the formation of blood vessels surrounding the implants. CD31 immunostaining and quantification of the density of CD31+ microvessels (MVD) revealed CC-Exos reduced angiogenesis when compared to the control. On the other hand, angiogenesis was promoted in the BMSC-Exos group. Green rectangles: typical CD31-positive vessels, which are in high magnification. In these images, CD31+ are red, and nuclei are blue (DAPI). “V”: CD31-positive blood vessels. Scale bars = 50 μm. **e** Western blot of VEGF, and SDF-1 secreted by the implants exposed to different exosomes and PBS

The effect of CC-Exos on promoting chondrogenesis of CPCs was evaluated in subcutaneous non-chondrogenic sites. At week 4, the CPC constructs supplied with PBS did not lead to noticeable cartilage matrix deposition. The addition of CC-Exos improved the matrix formation, which demonstrated similar morphological characteristics to those in native cartilage, with chondrocytes located within typical chondrocytes lacunae and surrounded by abundant cartilaginous matrix (Fig. 2). In addition, BMSC-Exos, as expected, also improved the cartilage regeneration of the CPC constructs. By 12 weeks, the BMSC-Exos and CC-Exos groups could maintain their cartilage-like appearance, and the histological results revealed significantly more contiguous cartilage matrix deposition than was observed at 4 weeks in each group. The new cartilage tissue exhibited intense staining of both S-F and T-B and showed cartilage-like tissue with typical histological structure and specific matrix deposition. More intense staining was noted in the CC-Exos and BMSC-Exos groups compared with the PBS group. In addition, the expression of typical cartilage markers such as COL II and SOX-9 was significantly enhanced in the BMSC-Exos and CC-Exos groups after 12 weeks (Fig. 3a, b), which indicated superior matrix formation in the implants.

### CC-Exos are conducive to maintain the phenotypic stability of engineered cartilage

We next explored whether the two kinds of exosomes were sufficient to steer the chondrogenesis of engineered tissues toward permanent cartilage-like tissues. Importantly, CPC constructs in the CC-Exos groups stained negatively for COL X (Fig. 3a), indicating tissue hypertrophy. However, implants in the BMSC-Exos group stained intensely for COL X. It is noteworthy that the hypertrophic cartilage-enriched markers of COL X, MMP 13, and IHH were strongly upregulated under long-term use of BMSC-Exos compared with CC-Exos (Fig. 3c). These observations suggested that CC-Exos are conducive to maintain the phenotypic stability of engineered tissue compared with BMSC-Exos.

Leijten reported that hypertrophic differentiation and subsequent calcification are associated with vascular invasion [44]. The angiogenesis in the engineered tissues was then investigated. In gross, the implants in the BMSC-Exos group appeared macroscopically vascularized, whereas CC-Exos implants were predominantly avascular, with some tiny blood vessels visible only in discrete peripheral regions (Fig. 3d). In fact, CD31 staining demonstrated the devoid presence of blood vessels in CC-Exos group. However, the MVD value (microvessels/hotspot) in the BMSC-Exos group was the highest among the three groups (*P* < 0.05, Fig. 3d). These results were further confirmed by the expression of angiogenic hallmarks, such as SDF-1 and VEGF, which were higher in samples from the BMSC-Exos group than those from the CC-Exos group (Fig. 3e).

### Exosome uptake and promote CPC proliferation and migration

To strengthen our in vivo findings, we next analyzed the underlying mechanism in vitro by assessing the effect of exosomes on the migration, proliferation, and matrix synthesis of CPCs.

**Fig. 4:**
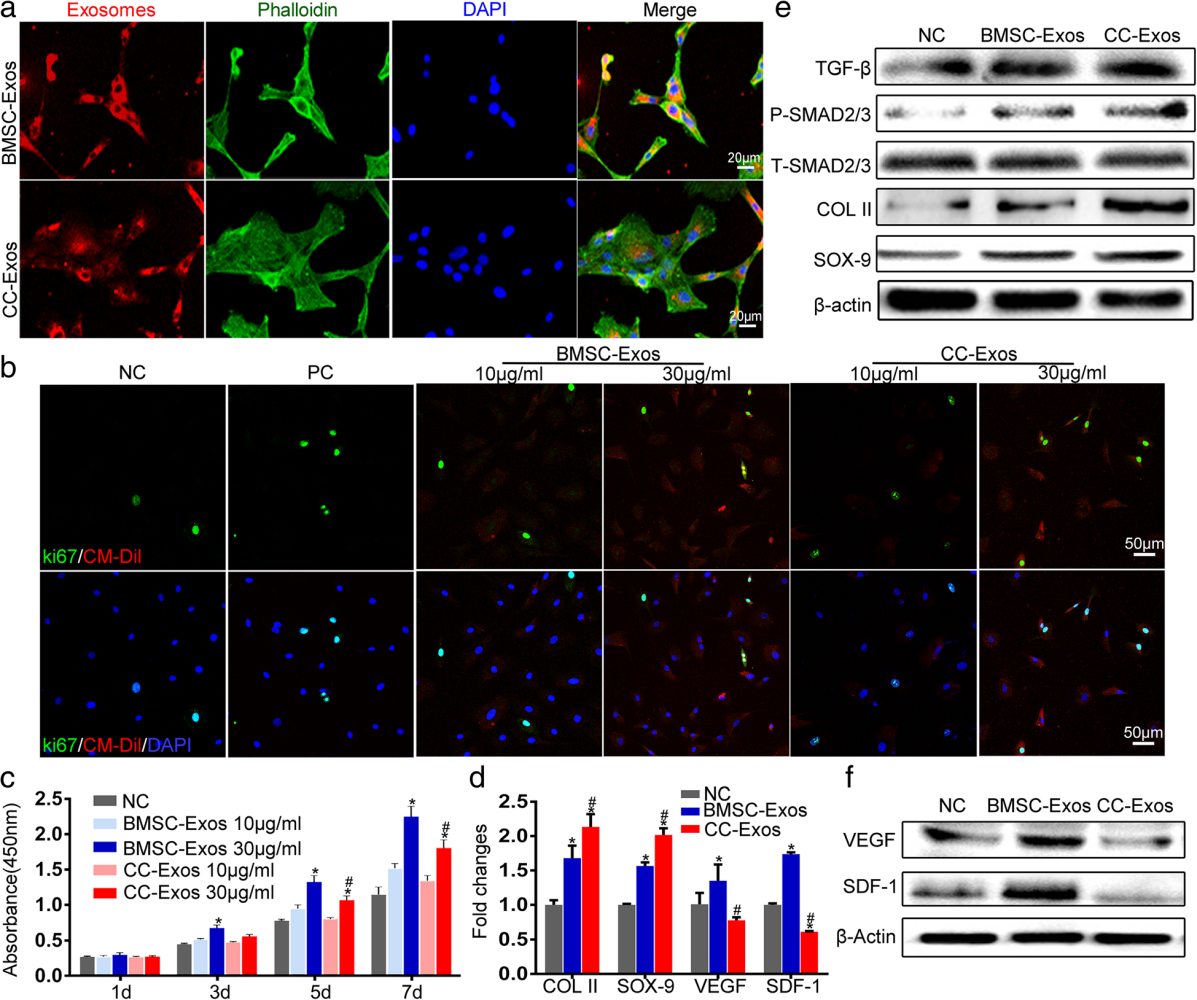
Effects of BMSC-Exos and CC-Exos on the proliferation and differentiation of CPCs. **a** Immunofluorescence staining revealed that there was no difference between the two kinds of exosomes internalized by CPCs. Exosomes are red (CM-Dil) and overlaid on cultures of proliferating CPCs for 12 h. Scale bar = 20 μm. **b** Immunofluorescence of Ki67 in CPCs incubated with BMSC-Exos and CC-Exos (10 μg/mL and 30 μg/mL) for 12 h. CPCs were stained for Ki67 and DAPI (nucleus) and considered positive when Ki67 overlapped the nucleus. In these images, Ki67 are green, exosomes are red (CM-Dil), and nuclei are blue (DAPI). Scale bar = 50 μm. **c** Proliferation of CPCs after BMSC-Exos and CC-Exos stimulation and the negative control for 1, 3, 5, and 7 days. **d** Gene analysis for chondrogenesis and angiogenesis of CPCs stimulated by different exosomes. qRT-PCR results demonstrated that the CC-Exos group showed the highest expression of cartilage-associated genes, such as COL II and SOX-9, and the angiogenesis-associated genes, such as VEGF and SDF-1. **e** Western blot analysis of TGF-β, P-SMAD2/3, T-SMAD2/3, COL II, and SOX-9 secreted by CPCs exposed to exosomes for 48 h. **f** Western blot analysis of angiogenesis-associated VEGF and SDF-1 secreted by CPCs exposed to exosomes for 48 h. Data are shown as the mean ± standard deviation for *n* = 3. *P < 0.05, compared to negative control (NC), ^#^*P* < 0.05, CC-Exos group compared to BMSC-Exos group

There was no difference between the two kinds of exosomes internalized by CPCs according to the mean fluorescence intensity of the same number of cells (Fig. 4a). At a concentration of 30 μg/mL, both BMSC-Exos and CC-Exos can stimulate CPC proliferation when compared to NC (*P* < 0.05, Fig. 4c). Furthermore, BMSC-Exos had a much stronger effect on CPC proliferation than CC-Exos (*P* < 0.05), whereas at a concentration of 10 μg/mL, there were no marked differences among the CC-Exos, BMSC-Exos, and NC groups (*P* > 0.05).

To further assess the different exosome-induced CPC proliferation, nuclear Ki67 staining was carried out as a surrogate for CPC proliferation (Fig. 4b). After incubating CPCs for 12 h on the substrates, 10 μg/mL of both BMSC-Exos and CC-Exos had a higher Ki67 expression than the negative control, which was more evident at 30 μg/mL. These results suggest that both CC-Exos and BMSC-Exos increase the mitogenic effect of the CPCs.

**Fig. 5:**
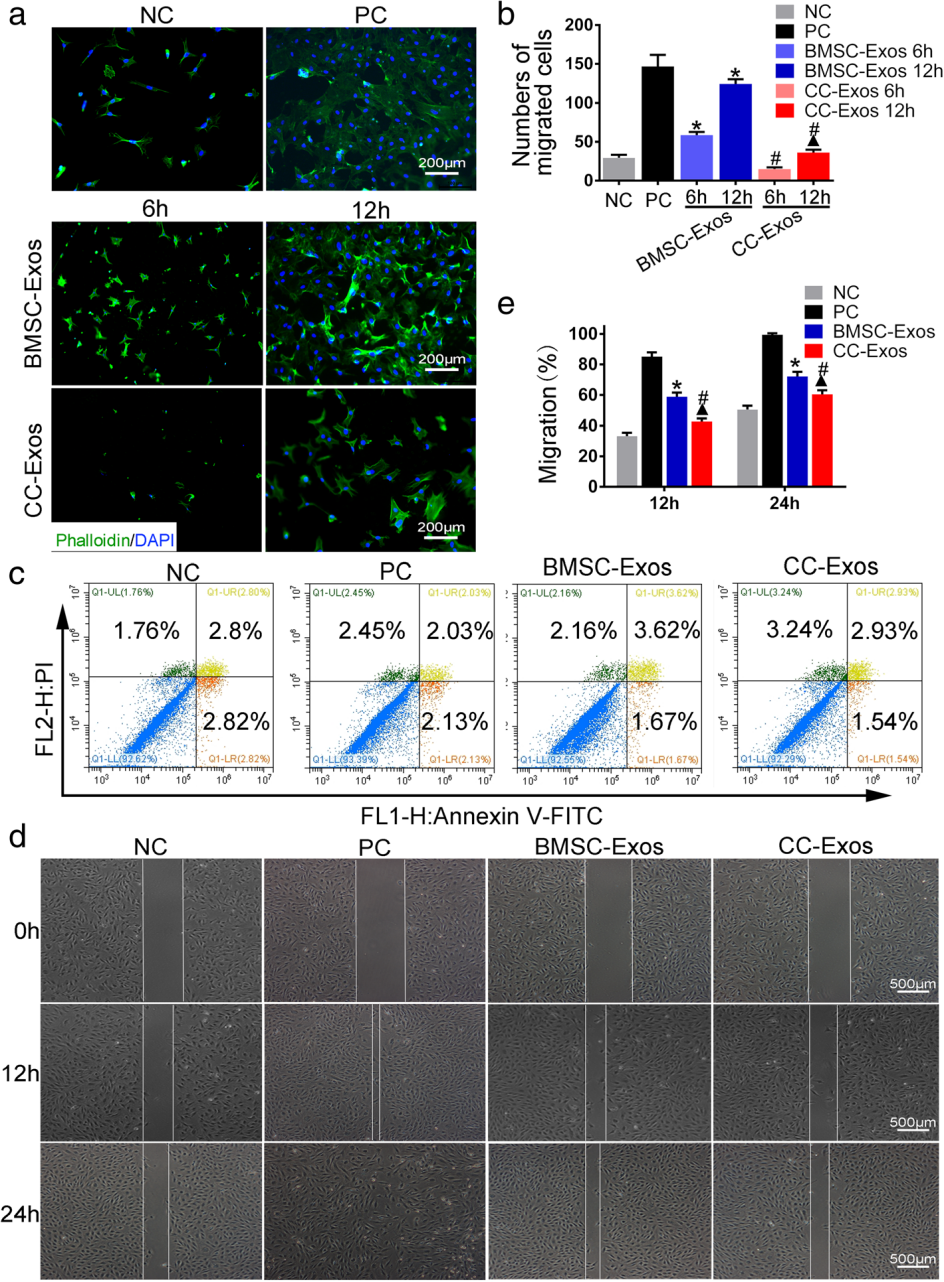
The effects of exosomes on CPCs migration. **a**, **b** Migration of CPCs exposed to different conditioned medium. Images of NC (negative control, DMEM medium with 1% FBS), PC (positive control, DMEM medium with 10% FBS), and CC-Exos and BMSC-Exos group (DMEM medium with 1% FBS and 30 μg/mL exosomes) show result of cell migration at 12 h and quantitative analysis of migrated cells. Exosomes are red, phalloidin is green, and nuclei are blue. Scale bar = 200 μm. **c** The apoptosis level of CPCs incubated with BMSC-Exos and CC-Exos for 12 h. Apoptotic cells were stained by PI/Annexin-V followed by flow cytometry. **d**, **e** Light microscopy images of the scratch wound assays and quantitative analysis of migration rates at 12 h and 24 h. Scale bar = 200 μm. *P < 0.05, compared to negative control (NC), ^▲^*P* < 0.05, compared to positive control (PC), ^#^*P* < 0.05, CC-Exos group compared to BMSC-Exos groups

An in vitro transwell assay was performed to investigate the exosome-stimulating effects of migration on CPCs. CC-Exos did not stimulate migration of CPCs after 12 h (*P* < 0.05, Fig. 5a, b). In contrast, CPC migration increased significantly in the presence of BMSC-Exos after 6 and 12 h, when compared with the NC or CC-Exos groups (*P* < 0.05), and the number of migrated cells approached the number identified in the PC group (*P* > 0.05). We also performed a cell apoptosis analysis at 12 h, which revealed no significant difference between the four groups (Fig. 5c). Based on these findings, we believe the different migration cell number was attributed to the different chemotaxis effect of the exosomes. Scratch wound assays further proved that BMSC-Exos were more effective than CC-Exos in increasing the motility of CPCs at 24 h (*P* < 0.05, Fig. 5d, e). The above results confirmed that the extracted BMSC-Exos and CC-Exos could promote the proliferation of CPCs, with BMSC-Exos exerting a stronger effect, while only BMSC-Exos can increase the migration of CPCs.

### CC-Exos promote the differentiation of CPCs

COL II and SOX-9 expression were then evaluated as indicators of chondrogenic differentiation. Importantly, when exposed to two kinds of exosomes, genes associated with chondrogenic differentiation and matrix synthesis, such as COL II and SOX-9, were significantly increased at the mRNA levels, with the CC-Exos exerting a stronger effect (*P* < 0.05, Fig. 4d). Since TGF-β signaling pathway is one of the main pathways to determine the chondrogenesis [45, 46], we also performed a preliminary study on the expression change of TGF-β signaling pathway after BMSC-Exos and CC-Exos treatment. CC-Exos stimulated the protein expression of TGF-β and SMAD2/3 (Fig. 4e). The downstream expression of COL II and SOX 9 were also increased. Thus, CC-Exos internalized by CPCs exerted chondrogenic effects possibly through the TGF-β/SMAD signaling pathway.

Furthermore, the factors involved in vascularization, such as VEGF and SDF-1, were significantly increased at the mRNA and protein levels in the BMSC-Exos group compared to the NC and CC-Exos group (*P* < 0.05, Fig. 4d, f). Interestingly, the mRNA levels of VEGF and SDF-1 in CPCs cultured with CC-Exos were lower than those in CPCs cultures with NC (*P* < 0.05), but the protein levels of the two groups had no discernible difference.

### BMSC-Exos and not CC-Exos promote HUVEC migration and angiogenesis

**Fig. 6:**
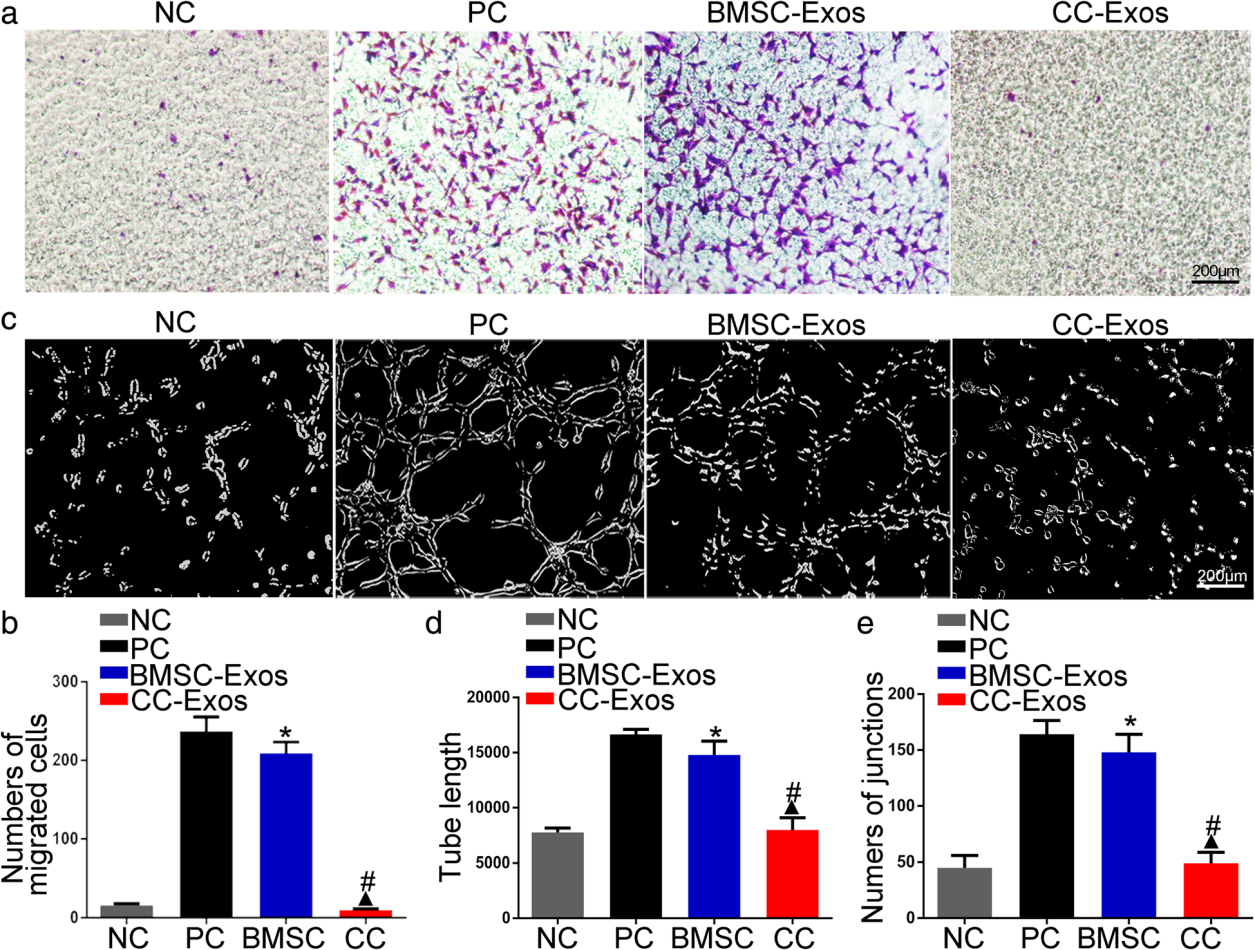
The effects of exosomes on HUVEC migration and tube formation. **a**, **c** Migration of HUVECs exposed to different exosomes for 6 h and a summary of the migrated cells. Cells are colored by violet, scale bar = 200 μm. **b**, **d**, **e**. HUVEC tube formation was studied by growing cells in Matrigel and quantitative analysis of total tube length and junction number after 3 h. *P < 0.05, compared to negative control (NC), ^▲^*P* < 0.05, compared to positive control (PC), ^#^*P* < 0.05, CC-Exos compared to the BMSC-Exos group

An in vitro transwell assay was performed to investigate the exosome-stimulating effects on HUVECs. HUVEC migration increased significantly in the presence of BMSC-Exos, compared to the NC and CC-Exos group (*P* < 0.05, Fig. 6a, b), and the number of migrated cells approached the number identified in the PC group (*P* > 0.05). However, CC-Exos had no significant effect on HUVEC migration, even compared with the NC group (*P* > 0.05).

Furthermore, the classical Matrigel assay demonstrated that BMSC-Exos exhibited the capacity to promote the formation of capillary-like structures compared with the NC or CC-Exos group (*P* < 0.05, Fig. 6c–e). Contrary to the angiogenic role of BMSC-Exos, the CC-Exos produced shorter networks with the least number of mesh, values that were not significantly different than the NC group (*P* > 0.05).

In short, these findings indicate that although BMSC-Exos could promote CPCs proliferation and migration, it was also prone to angiogenesis and hypertrophic differentiation. On the other hand, the CC-Exos displayed a robust chondral matrix formation with minor angiogenesis, which circumvented the inherent drawbacks of BMSC-Exos. The results further corroborated its preferable cartilage regeneration and stability of CC-Exos in vivo.

## Discussion

Cartilage defect treatment represents a major clinical problem worldwide because of the increasing incidences of trauma, disease, or aging [47, 48]. In clinics, autogenous cartilage is typically required to implant to the defect sites in order to restore normal appearance and function [49–51], which was fraught with a couple of shortcomings [5, 52, 53]. Tissue-engineered cartilage grafts have emerged as a promising alternative to overcome these problems and satisfy the ever-increasing clinical need [54–56]. Currently, exosomes have been identified as the principal agent in mediating the therapeutic efficacy of the cell-based regenerative medicine approach [20, 21, 57, 58], and BMSC-Exos have been reported for promoting in situ cartilage defect repair [21, 26]. However, the therapeutic outcome for subcutaneous cartilage defect repair is still limited because of the lack of a suitable pro-chondrogenic environment [17, 40, 59]. Meanwhile, previous studies have also shown that chondrocytes could steer the chondrogenesis of stem cells in vitro and in vivo through paracrine effects [1, 7, 60]. In the present study, using a CPC-based cartilage tissue engineering approach, the potential of CC-Exos in promoting ectopia chondrogenesis and stabilizing cartilage regeneration in a subcutaneous environment was further investigated.

The current study demonstrated that CPC constructs supplied with CC-Exos could form homogeneous cartilage-like tissue with minimal hypertrophy in a subcutaneous environment, with no help from any chondrogenic factors. Furthermore, a series of in vitro experiments further confirmed that CC-Exos significantly promoted chondrogenesis-related factors at the mRNA and protein levels in CPCs, such as SOX-9 and COL II. Importantly, angiogenesis was inhibited by CC-Exos, which is known to be detrimental to cartilage regeneration leading to hypertrophic differentiation and subsequent calcification [44]. The observed contributions of CC-Exos to cartilage regeneration in vivo corroborate the in vitro findings and further support that CC-Exos alone could provide a preferable chondrogenic environment and help maintain the stability of cartilage tissue. Compared with BMSC-Exos where samples show more hypertrophic cartilage, the cartilage regeneration results achieved with the use of CC-Exos are significantly more favorable. Hence, the null hypothesis that there is no difference between CC-Exos and BMSC-Exos in cartilage regeneration results must be rejected.

To date, tissue engineering has offered promising solutions for clinical issues involving congenital and acquired cartilage defects [7, 61, 62]. However, the cartilage formation in subcutaneous environments is limited due to the lack of a proper chondrogenic niche [11, 12, 59]. Imitating the chondrogenic niche is a well-accepted approach to promote the ectopic chondrogenesis of progenitor cells [16, 18]. Exosomes have been studied extensively for their potential in participating in the maintenance of normal physiology via delivering various types of bioactive microRNAs, nucleic acids, proteins, and unique gene products [22, 63]. Recent studies have shown that chondrocytes and chondrocyte-related factors play key regulatory roles in the maintenance of the cartilage microenvironment and the ultimate cartilage phenotype of implanted stem cells [13–15]. In the present study, we further demonstrate that CC-Exos modulates CPC migration, proliferation, and cartilage matrix synthesis. Expression of SOX-9 and COL II by CPCs is upregulated in the presence of CC-Exos, which promotes chondrogenesis. This may be attributed to the TGF-β/SMAD signaling pathway, which is reported to play an essential role in chondrocyte differentiation and matrix maturation [41, 45, 64]. More investigations are needed to acquire the whole picture of the pathway involved in CC-Exos-induced chondrogenesis.

Additionally, reproducibly generating stable cartilage remains an unsolved challenge. Avoiding vessel ingrowth and hypertrophy is a critical factor in building stable cartilage [65, 66]. In the present study, compared to the positive control groups (BMSC-Exos), CC-Exos could maintain a stabilized phenotype of constructed cartilage at least within the investigated time frame, as evidenced by the presence of significantly less COL X-positive staining and minimal protein expression of COL X, IHH, and MMP 13 secreted by hypertrophic chondrocytes at 12 weeks. In addition, less CD31-positive microvessels are observed in the neo-cartilage of the CC-Exos group. However, after the addition of BMSC-Exos, expression of SDF-1 and VEGF is upregulated, which promotes cell homing and angiogenesis. This is beneficial for cartilage engineering during the early stage of implantation [40], which may account for the better neo-cartilage formation in the BMSC-Exos, as reported [21, 26]. However, it also has a disadvantage as evidenced by promoting associated ectopic cartilage hypertrophy. Recent studies have also shown that vascular invasion is one of the major mechanisms involved in hypertrophic cartilage differentiation [5, 44]. In vitro results also revealed that HUVEC migration and tube formation are reduced by CC-Exos when compared with BMSC-Exos. These results collaborate with data collected from an in vivo experiment, which shows CC-Exos have the ability to decrease angiogenesis in subcutaneous cartilage repair. Because CC-Exos can promote CPC migration, proliferation, and matrix synthesis in vitro, a more favorable prognosis is anticipated for long-term cartilage regeneration.

A novel method of imitating the chondrogenic niche is explored in the present work via the use of CC-Exos. After local injection of CC-Exos, the CPCs are rapidly directed to form neo-cartilage, when stimulated by chondroinductive mediators. Importantly, the engineered cartilage here can maintain the stabilized phenotype in non-chondrogenic niches, which is probably related to antiangiogenic factors secreted by CC-Exos that prevent neovascularization and hypertrophy. Because strategies that provide the conventional cartilage environment often require cell-based therapy [54, 55], the use of CC-Exos is advantageous from the perspectives of off-the-shelf and cell-free regenerative medicine approach for cartilage repair, and the ease of minimally invasive injection of CC-Exos concentrate.

Despite these encouraging results, the exact component(s) is yet to be elucidated. It is plausible that a myriad of components is present in the CC-Exos that can orchestrate cartilage regeneration including chondrogenesis and stability. However, the detailed mechanism of CC-Exos treatment to CPCs that caused the difference from that of BMSC-Exos is still unclear, and further investigation of RNA-seq is needed to dissect the components present in CC-Exos and to investigate their underlying mechanisms in cartilage repair. In addition, chondrogenesis is a complex process which is related to various signaling pathways, such as wnt, TGF-β, and hedgehog pathway. Here, we preliminarily demonstrated the induction of TGF-β and downstream SMAD2/3 expression after CC-Exos treatment. Further investigation is needed to acquire the entire picture of the pathway.

## Conclusions

In summary, this study demonstrated that a novel exosome from chondrocytes could imitate the chondrogenic niche in a subcutaneous environment, which could facilitate chondrogenesis and maintenance of cartilage stability. This may contribute to its preferable chondroinductive niches coupled with its antiangiogenic properties. Thus, CC-Exos may represent a promising biologic-based therapeutic approach for the treatment of ectopic cartilage defects.

## Abbreviations

BMSC: Bone mesenchymal stem cells
BMSC-Exos: Exosomes secreted by bone mesenchymal stem cells
CC-Exos: Exosomes secreted by chondrocytes
COL II: Collagen type II
COL X: Collagen type X
CPCs: Cartilage progenitor/stem cells
DMEM: Dulbecco’s modified Eagle’s medium
FBS: Fetal bovine serum
H&E: Hematoxylin and eosin
HUVECs: Human umbilical vein endothelial cells
IHH: Indian hedgehog
MMP 13: Matrix metalloproteinase 13
NC: Negative control
PBS: Phosphate-buffered saline
PC: Positive control
RT-PCR: Real-time quantitative polymerase chain reaction
SDF-1: Stem cell-derived factor 1
S-F: Safranin-O/fast green
T-B: Toluidine blue
TEM: Transmission electron microscopy
TGF-β: Transforming growth factor-β
VEGF: Vascular endothelial growth factor
